# Impact of focused cardiac and lung ultrasound screening performed by a junior doctor during admission to the surgical ward on patients before emergency non‐cardiac surgery: A pilot prospective observational study

**DOI:** 10.1002/ajum.12321

**Published:** 2022-10-13

**Authors:** Cliff Wong, Rukman Vijayakumar, David J Canty, Colin F Royse, Yang Yang, Alistair G Royse, Johan Heiberg

**Affiliations:** ^1^ Department of Surgery University of Melbourne Melbourne Victoria Australia; ^2^ Department of Anaesthesia and Pain Management Royal Melbourne Hospital Parkville Victoria Australia; ^3^ Department of Anaesthesia and Perioperative Medicine Monash Health Clayton Victoria Australia; ^4^ Department of Medicine Monash University Clayton Victoria Australia; ^5^ Outcomes Research Consortium Cleveland Clinic Cleveland Ohio USA; ^6^ Intensive Care Unit Western Hospital Footscray Victoria Australia; ^7^ Department of Cardiothoracic Surgery Royal Melbourne Hospital Parkville Victoria Australia; ^8^ Department of Anaesthesia, Centre of Head and Orthopaedics Copenhagen University Hospital, Rigshospitalet Kobenhavn Denmark

## Abstract

**Purpose:**

To assess whether pre‐operative focused cardiac ultrasound and lung ultrasound screening performed by a junior doctor can change diagnosis and clinical management of patients aged ≥65 years undergoing emergency, non‐cardiac surgery.

**Method:**

This pilot prospective observational study included patients scheduled for emergency, non‐cardiac surgery. The treating team completed a diagnosis and management plan before and after focused cardiac and lung ultrasound, which was performed by a junior doctor. Changes to diagnosis and management after ultrasound were recorded. Ultrasound images were assessed for image and diagnostic interpretation by an independent expert.

**Results:**

There was a total of 57 patients at age 77 ± 8 years. Cardiopulmonary pathology was suspected after clinical assessment in 28% vs. 72% after ultrasound (including abnormal haemodynamic state in 61%, valvular lesions in 32%, acute pulmonary oedema/interstitial syndrome in 9% and bilateral pleural effusions in 2%). In 67% of patients, the perioperative management was changed. The changes were in fluid therapy in 30%, cardiology consultation in 7%, formal in‐ or out‐patient, transthoracic echocardiography in 11% and 30% respectively.

**Discussion:**

The impact of pre‐operative focused cardiac and lung ultrasound on diagnosis and management of patients on the hospital ward before emergency non‐cardiac surgery by a junior doctor was comparable to previous studies of anaesthetists experienced in focused ultrasound. However, the ability to recognise when image quality is insufficient for diagnosis is an important consideration for novice sonographers.

**Conclusions:**

Focused cardiac and lung ultrasound examination by a junior doctor is feasible and may change preoperative diagnosis and management in patients of 65 years or older, admitted for emergency non‐cardiac surgery.

## Introduction

A thorough pre‐operative evaluation and optimisation of patients' comorbidities prior to surgery is important to reduce perioperative mortality.^1^ However, in the setting of emergency surgery there may be insufficient time or resources for definitive cardiac investigations such as transthoracic echocardiography, myocardial perfusion studies or cardiopulmonary exercise testing. As a result, surgery may proceed without complete medical assessment, which may expose patients to risk of cardiopulmonary decompensation. Point‐of‐care cardiac and lung ultrasound has been embraced by anaesthetists and critical care physicians^2^, ^3^, ^4^ as it may be performed at short notice at the patient's bedside resulting in improved diagnostic accuracy with little, if any, risk of surgical delay or need for transport to radiology or echocardiography departments. There is evidence that pre‐operative focused cardiac and lung ultrasound changes the diagnosis and management. In a systematic review by Heiberg *et al*.,^5^ when focused echocardiography was performed in patients at risk of cardiac disease, including the elderly, diagnostic changes occurred between 51% and 67% of cases and changes in management between 54% and 82%. In the studies included in the systematic review, focused cardiac ultrasound was performed by consultant‐level anaesthetists with substantial training in echocardiography. However, not all anaesthetists are trained in ultrasound, and hence, the number of patients who can be assessed is limited, and if junior doctors performed focused cardiac and lung ultrasound more widely, it may greatly increase the volume of patients receiving the benefits. As current available guidelines regarding competency requirements are heterogenous,^6^ studies assessing the impact of non‐expert performed point‐of‐care ultrasound in the perioperative setting are highly warranted.

The aim of this pilot study was to determine whether routine screening for cardiac and respiratory disease by a junior doctor with focused cardiac and lung ultrasound changes the diagnosis and management in patients undergoing emergency non‐cardiac surgery. We hypothesised that focused ultrasound examination performed by a junior doctor would alter patient diagnosis and management.

## Material and methods

### Design and study participants

In this prospective observational study, participants admitted to the Royal Melbourne Hospital were identified and screened for eligibility using the hospital's electronic emergency surgery booking database and eligible patients were assessed for inclusion by a junior researcher who was not responsible for the medical care of the patient. Inclusion criteria were patient aged 65 years or above and scheduled for emergency non‐cardiac surgery. Emergency surgery was defined as surgery required within 48 h and non‐elective admission. Exclusion criteria included results available for either resting or stress transthoracic echocardiogram, cardiac nuclear medicine scan or chest computed tomography performed within 12 months of screening. As this was a pilot study to determine the feasibility of junior doctor‐performed ultrasound on changes to diagnosis and management in the pre‐operative setting, a convenience sample size of 50 patients was selected to inform future sample size and provide preliminary evidence of efficacy.

### Data collection

A standard assessment was performed by the treating surgical team comprising of clinical history, physical examination and standard investigations such as serum biochemistry, haematology, blood gas analysis, electrocardiogram and chest x‐ray. The treating surgical team was asked to complete a research form (Appendix S1) detailing their clinical diagnosis and treatment plan for the patient. A junior medical officer from the research team (RV), who was blinded to the medical history of the participant and the above diagnosis and management form, then performed focused cardiac and lung ultrasound examination according to the protocol described below. Prior to the commencement of the study, the junior doctor had received supervised training in focused cardiac and lung ultrasound, including 30 supervised examinations of patients with pathology, described in a previous study^7^ and Table S1. The ultrasound findings were reported on a standardised report form (Figure S1) and communicated to the treating team. Lastly, the treating surgical team was asked to complete a second diagnosis and management plan following information from the ultrasound findings. As the pre‐ and post‐ultrasound diagnosis and management forms were completed immediately before and after the ultrasound, any changes in treatment were recorded as a result of the ultrasound examination. Images acquired from the junior doctor's ultrasound examination were later reviewed by an expert sonographer for assessment of image acquisition and interpretation. The expert's review was not communicated to the treating surgical team, and therefore, management changes occurred solely on the basis of the junior doctor's ultrasound findings and at the discretion of the treating surgical team only. A graphical overview of the data collection process is shown in Figure S2.

### Ultrasound protocol

Ultrasound was performed using a hand‐held ultrasound system, SonoSite iViz (FUJIFILM, Bothell, WA, USA) with a phased array 1–5 MHz transducer, capable of colour flow Doppler and 2D measurements but not spectral Doppler. The focused cardiac and lung ultrasound examinations were conducted according to the iHeartScan^8^, ^9^, ^10^, ^11^, ^12^, ^13^, ^14^, ^15^ and iLungScan^9^, ^11^, ^16^ protocols, which have been reported and validated in the perioperative setting. These protocols are designed to take less than 10 min each to perform using pattern recognition of two‐dimensional and colour flow Doppler images, enabling convenient point‐of‐care use.

To determine haemodynamic state, the following components were assessed and reported: (i) Left ventricular volume, (ii) Left ventricular systolic function and (iii) Left atrial filling pressure based on interatrial septum movement.^10^ Sonographic definitions for the components of haemodynamic state assessment are described in Table 1. Based on these components, the overall haemodynamic state was categorised as (i) normal, (ii) fluid responsive state (i.e. hypovolaemic or vasodilated) and (iii) heart failure (i.e. primary systolic dysfunction, primary diastolic dysfunction, or combined systolic and diastolic dysfunction) as described Royse *et al*.^16^ (Table 2).

**Table 1:** Components assessed and definitions of abnormal findings in focussed cardiac ultrasound.

Component	Definitions
LV volume	LVEDD: Normal: 3–5.6 cm; Dilated: >5.6 cm; Empty: <3 cm OR LVEDA: Normal: 8–14 cm^2^, Dilated >14 cm^2^; Empty: <8 cm^2^
LV systolic function	Fractional shortening: Normal: 28–44%; Reduced: <28%; Increased: >44% Fractional area change: Normal: 50–65%; Reduced: <50%; Increased: >65%
LA filling pressure	Normal: Systolic reversal of interatrial septum, throughout cardiac cycle High: Fixed curvature of the interatrial septum away from LA Low: Systolic bucking of the interatrial septum
Significant aortic stenosis	An opening <15 mm in Parasternal Long Axis OR Heavy calcification of aortic valve with inability to see valve opening
Significant aortic regurgitation	A jet that runs on the wall of left ventricular outflow tract OR A jet that is wider than 25% of the diameter of left ventricular outflow tract OR A jet that extends down to the ventricle (over 2.5 cm long)
Significant mitral stenosis	Impaired opening of mitral valve OR hockey stick appearance of mitral leaflets
Significant mitral regurgitation	Regurgitation jet covering >20% of the left atrium area in A4C or PLAX OR A turbulent jet that runs along the wall of the atrium OR Prominent flail mitral valve leaflet or ruptured papillary muscle
Significant tricuspid regurgitation	Any edge‐tracking jet OR Any central Jet >5 cm^2^

A4C, apical 4 chamber view; FCU, focused cardiac ultrasound; LA, left atrium; LV, left ventricle; LVEDA, left ventricular end‐diastolic area measured in parasternal short axis at the mid‐ventricle level; LVEDD, left ventricular end‐diastolic diameter measured in parasternal long axis at the base of the left ventricle; LVESA, left ventricular end‐systolic area; PLAX, parasternal long axis view.

Fractional shortening = (left ventricular end‐diastolic diameter−left ventricular end‐systolic diameter)/left ventricular end−diastolic diameter.

Fractional area change = (left ventricular end‐diastolic area−left ventricular end‐systolic area)/left ventricular end−diastolic area.

**Table 2:** Definitions of overall haemodynamic state.

	Left ventricular volume	Left ventricular systolic function	Left atrial filling pressure
Normal	Normal	Normal	Normal
Hypovolaemia	Decreased	Normal/Decreased	Decreased
Vasodilated	Normal	Increased	Normal
Primary systolic dysfunction	Increased	Decreased	Normal
Primary diastolic dysfunction	Normal/Decreased	Normal	Increased
Systolic and diastolic dysfunction	Increased	Decreased	Increased

Identification of clinically significant valvular lesions was performed by observing leaflet appearance, and thickness and opening for stenotic lesions while regurgitant lesions^17^, ^18^ were assessed by the presence and severity of a reverse jet using colour flow doppler. Sonographic definitions for clinically significant valvular lesions used in this study can be found in Table 1.

For the lung ultrasound examination, the procedure was standardised and followed the iLungScan protocol as established by the University of Melbourne, Ultrasound Education Group.^19^ Patients were in a semi‐recumbent position at 30 degrees head up for the examination, which was performed in 3 anatomical zones of each lung. A normal lung pattern was identified by the presence of normal lung sliding or lung pulse, reverberation artefacts from the pleura and absence of the following pathologies^20^: (i) Collapse or atelectasis pattern, defined by a loss of lung volume, increased tissue density and hyperechoic static air bronchograms,^21^ (ii) Consolidation defined by a tissue‐like pattern or ‘hepatisation’ with minimal volume loss and the presence of dynamic air bronchograms in affected lung,^22^, ^23^ (iii) Alveolar‐interstitial syndrome defined as 3 or more B‐lines in a single rib space^23^, ^24^ with B‐lines being defined strictly as hyperechoic, vertical artefacts arising from the pleural line that move with lung sliding and reach the bottom of the screen without fading while ablating reverberation artefacts from the pleura,^25^ (iv) Pleural effusion defined as a space between the parietal and visceral pleura with movement with the respiratory cycle,^23^ and (v) Pneumothorax defined as the absence of lung sliding and lung pulse.^26^

### Assessment of image acquisition and interpretation

All images acquired during the study were stored on a secure cloud‐based server for assessment of image quality and interpretation by an independent expert (YY) qualified and experienced in focused cardiac and lung ultrasound and blinded to the patient's clinical information.

The focused cardiac and lung ultrasound images recorded by the junior doctor (RV) were reviewed by this expert, and an interpretive report was completed for each patient. The report provided by the expert was not communicated to the treating surgical team and was used exclusively for assessment of image interpretation.

Assessment of image interpretation for focused cardiac ultrasound was divided into (i) assessment of haemodynamic state and (ii) identification of clinically significant valvular lesions. The junior doctor's interpretation was considered accurate when all components were identical to the interpretation provided by the independent reviewer (YY). If images acquired by the junior doctor were of insufficient quality for the independent expert to comment on haemodynamic state or valvular dysfunction, the images were excluded from analysis.

For assessment of lung ultrasound image interpretation, the expert interpreted as either presence or absence of collapse/consolidation, interstitial syndrome/acute pulmonary oedema, pneumothorax and/or pleural effusion as described above. For each pathology, the junior doctor's interpretation was considered accurate when identical to the interpretation provided by the independent observer.

Image quality assessment was performed using a previously described image quality scoring system^7^ (Appendices S2 and S3). This scoring system was initially designed to apply to the comprehensive transthoracic echocardiogram (TTE) protocol of 11 views but has been modified to include only four ‘core’ views, which are usually sufficient to assess all of the components of the iHeartScan‐focused assessment.^9^ These ‘core’ views are the parasternal long axis view, parasternal short axis mid‐ventricle view, apical four‐chamber view and subcostal inferior vena cava view. For each of the four ‘core’ views, a score was given by the expert according to this scoring system and an acceptable image quality was defined as greater than 64%, as defined in a previous study.^7^ Image quality of lung ultrasound was not performed as at that time a practical lung ultrasound image quality scoring system was not available. If no image was acquired by the junior doctor, it was excluded from the analysis.

### Outcomes

The primary outcome was any change in cardiac or respiratory diagnosis due to ultrasound examination performed by the junior doctor. Secondary outcomes assessed any changes in management plan due to ultrasound examination. Other secondary outcomes included quality of image acquisition and interpretation of focused cardiac and lung ultrasound performed by the junior doctor.

### Statistical analyses

Data on diagnosis and patient management are presented as percentages of patients, and data on image quality are presented as median image quality scores with interquartile range. To assess estimates of interobserver agreement between a junior doctor and an expert reviewer beyond that expected by chance, Cohen's Kappa (k) statistics was used. Values of k < 0.20 indicated poor strength of agreement, k = 0.21–0.4 fair strength of agreement, k = 0.41–0.60 moderate strength of agreement, k = 0.61–0.80 good strength of agreement and k = 0.81–1.0 very good strength of agreement. For this secondary outcome measure, p values<0.05 were considered statistically significant. Only images deemed of acceptable image quality by the expert reviewer were included. As this observational study was a pilot study, group comparisons were not performed. Descriptive data were stored in Microsoft Excel 2016 (Microsoft Corp., Redmond, WA, USA), and for statistical analyses, we used Stata/IC 15.1 for Mac (Stata Corp., College Station, TX, USA).

### Ethics approval

The Human Resources and Ethics Committee from the Royal Melbourne Hospital approved the study (LNR/16/MH/91), which conforms to the ethical guidelines of the 1975 Declaration of Helsinki. Prior written informed consent was obtained from all participants between June 2017 and October 2018.

## Results

**Figure 1:**
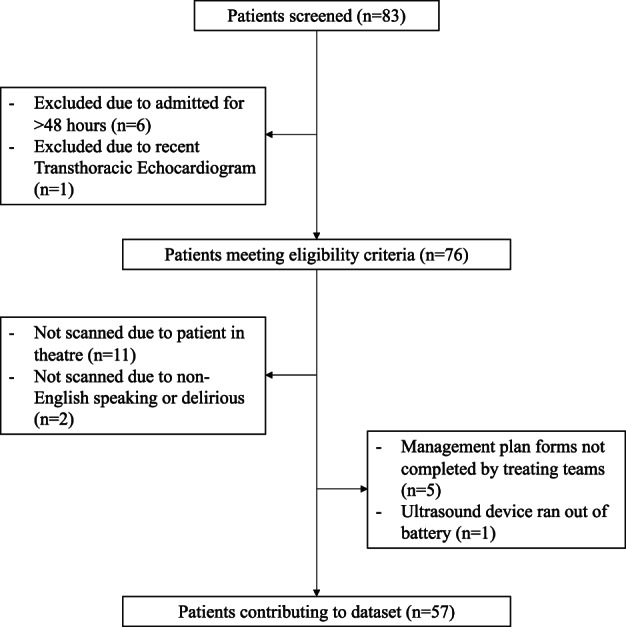
Patient inclusion.

In the period between June 2017 and October 2018, 83 patients were screened for eligibility, and 76 patients met the criteria of which 57 patients had complete datasets as displayed in Figure 1. Mean patient age at enrolment was 77 ± 8 years and included a wide range of surgical specialties including orthopaedic, neuro, vascular, general (abdominal), plastic, urology, endoscopy and ophthalmologic surgery (Table 3).

**Table 3:** Baseline characteristics of patients undergoing emergency, non‐cardiac surgery.

Demographics	N = 57
Age, years	77 ± 8
Male sex, n (%)	28 (49)
Hypertension, n (%)	28 (49)
Previous myocardial infarction, n (%)	9 (16)
Previous coronary intervention, n (%)	8 (14)
Congestive heart failure, n (%)	6 (11)
Atrial fibrillation, n (%)	7 (12)
Previous valvular replacement, n (%)	2 (4)
Chronic obstructive pulmonary disease, n (%)	8 (14)
Diabetes, n (%)	2 (4)
Renal failure, n (%)	6 (11)
Previous stroke, n (%)	2 (4)
Pulmonary hypertension, n (%)	0 (0)
Specialty	
Orthopaedics n (%)	23 (39)
Neurosurgery, n (%)	9 (16)
Vascular surgery, n (%)	8 (14)
General surgery, n (%)	7 (12)
Plastic surgery, n (%)	4 (7)
Urology	4 (7)
Gastroenterology	2 (4)
Ophthalmology	1 (2)

Data reported as means ± standard deviations or absolute numbers and percentages of patients.

Congestive cardiac failure was defined as signs or symptoms of dyspnoea or fatigue, orthopnoea, paroxysmal nocturnal dyspnoea, increased jugular venous pressure, pulmonary rales on physical examination, cardiomegaly or pulmonary vascular engorgement and renal disease was defined according to the RIFLE criteria.

### Impact on diagnosis and peri‐operative management

**Figure 2:**
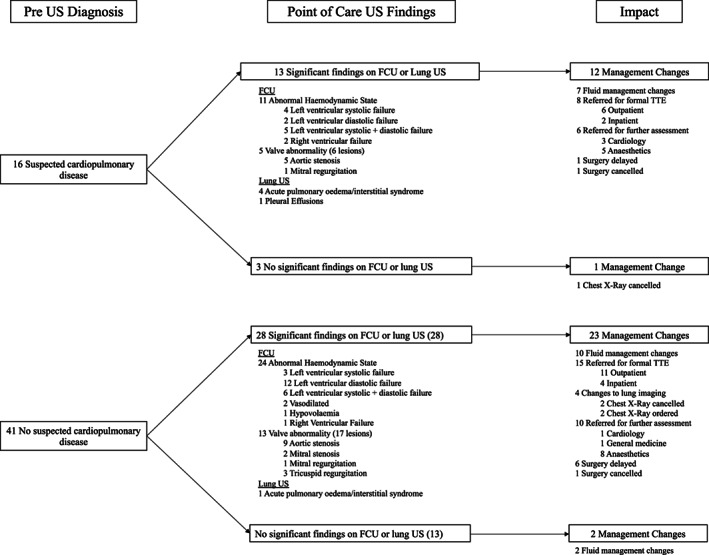
Summary of findings of clinical assessment, pre‐operative ultrasound and changes to diagnosis and management plans in 57 emergency surgery patients. FCU, focused cardiac ultrasound; TTE, transthoracic echocardiogram; US, ultrasound. Numbers may not add up as patients may have had more than one diagnosis or change in management.

The impact of ultrasound performed by a junior doctor on diagnosis and management is summarised in Figure 2 and Table S2. Clinical (standard) assessment before ultrasound reported that 28% of the patients were suspected of having either significant cardiac or respiratory disease. Focused ultrasound of the heart and lungs led to a change in management in 67% of patients. In the 41 (72%) patients assessed as being normal with clinical assessment, 28 (68%) patients were identified to have significant disease with ultrasound that led to a management change in 23 (82%) patients. In the 16 patients where disease was suspected on clinical assessment, ultrasound either confirmed the presence of disease or identified a different diagnosis to clinical assessment in 13 (81%) patients, leading to a management change in 12 patients. Ultrasound reassured the surgical team of 3 (5%) patients in whom the clinical diagnosis of pathology was downgraded to non‐significant or normal diagnosis by ultrasound. The most common pathologies identified with ultrasound were haemodynamic abnormality (61%), valvular abnormality (32%) and acute pulmonary oedema (9%). The most common changes in management were changes to fluid therapy (30%) and pre‐operative anaesthetics review (22%), followed by ordering of a formal inpatient TTE (11%) and referral to cardiology (7%) prior to surgery. Ultrasound resulted in a delay in surgery in 7 patients (12%) and cancellation in 2 (4%) patients. An additional 30% of the patients had formal out‐patient TTE requested.

### Quality of image acquisition and interpretation

Image quality scores for focused cardiac ultrasound are shown in Table 4. The median overall image quality score was 23 (17–26) of 31(74%), above the *a‐priori* minimum acceptable image quality score of 64%. The lowest image quality score was in the apical 4‐chamber view with median score of 6 (4–7) out of 9. The highest score was in the parasternal long axis view with a median of 8 (6–9) out of 10.

**Table 4:** Image quality scores for the focused cardiac ultrasound views.

Focused cardiac ultrasound view	Patients with a view obtained (N = 57)	Maximum score	Score
Parasternal long axis	49	10	8 (6–9)
Parasternal short axis (mid‐ventricle)	37	8	6 (5–7)
Apical 4‐chamber	46	9	6 (4–7)
Subcostal inferior vena cava	37	4	3 (2–3)
Overall image quality score	‐	31	23 (17–26)

Scores are presented as medians with interquartile ranges.

Of the 57 patients enrolled in the study, Forty patients had saved imaging deemed of acceptable quality by the independent reviewer for assessment of haemodynamic state interpretation, whereas only 18 patients had imaging with acceptable quality for assessment of valvular interpretation. For lung ultrasound, 22 patients had imaging with acceptable quality for assessment of the interpretation of lung ultrasound findings (6 unacceptable quality, 29 not scanned).

Interobserver agreements beyond chance, for assessment of the haemodynamic component, valvular component and lung component were assessed using Cohen's Kappa statistic. For the identification of clinically significant valvular disease (n = 18), Cohen's Kappa was 0.55 (95% CI: 0.09–1.00, P = 0.001), and for the lung ultrasound component (n = 22), Cohen's Kappa was 0.83 (95% CI: 0.42–1.00, P < 0.001). For the overall haemodynamic state (n = 43), Cohen's Kappa was 0.065 (95% CI: −0.12–‐0.26, P = 0.27). A normal haemodynamic state identified by the expert sonographer was misinterpreted as abnormal by the junior doctor in 28/43 patients (65%), while only one patient with abnormal haemodynamic state was missed.

## Discussion

In this observational study, we showed that focused cardiac and lung ultrasound examination performed by a junior doctor on patients presenting for emergency non‐cardiac surgery resulted in frequent changes in diagnoses and management compared with standard pre‐operative clinical assessment. When clinically important abnormalities were diagnosed on ultrasound, the predominant effects were adjustments in fluid administration, involvement of other inpatient units in the perioperative optimisation of patients and ordering of either chest x‐ray or conventional transthoracic echocardiography.

A number of studies have previously described a high impact of pre‐operative ultrasound on cardiac and lung ultrasound diagnoses^13^, ^27^, ^28^, ^29^ when performed immediately before urgent non‐cardiac surgery. In our study, significant findings were identified with focused ultrasound in 68% (28/41) of patients where there was no suspicion of cardiopulmonary disease by conventional examination and a change in management occurred in 67% (38/57) patients. These results are comparable to a systematic review by Heiberg *et al*.^5^ on pre‐operative focused cardiac ultrasound in patients at risk of cardiac disease, including the elderly, where diagnostic changes occurred between 51% and 67% of cases and changes in management between 54% and 82%. In the previous reports, focused cardiac ultrasound was performed by consultant‐level anaesthetists, and therefore, the impact on management was predominantly on haemodynamic strategy, anaesthetic technique, level of intraoperative and post‐operative care and surgical flow. In our study, where the ultrasound was performed earlier, the predominant impact on management was adjustment to intravascular fluid therapy, request for confirmatory conventional TTE, referral to other inpatient teams, and in some patients resulting in delay or cancellation of surgery. Lung ultrasound only contributed to a minority of changes in diagnosis and management. This might be reflective of a lower frequency of lung pathology in patients admitted for non‐cardiac surgery compared with cardiac and thoracic surgery.^19^

The mean image quality score in this study was above the acceptable lower limit of 64%.^7^ The high overall image quality score of 74% in this study may reflect that only the four ‘core’ views were included, but these views are sufficient to assess all of the variables required for the iHeartScan assessment. In combination, these views are likely to provide adequate imaging to determine features of cardiac failure and hypovolemia, to assess the aortic, mitral and tricuspid valves, and to determine the need for formal imaging and pre‐operative assessment by other inpatient units. In our assessment of image interpretation, the interpretation of lung ultrasound by the junior doctor had high diagnostic accuracy and agreement with the expert reviewer while the valve component of focused cardiac ultrasound had moderate accuracy and agreement, beyond what can be expected by chance. Importantly, undiagnosed valve lesions are common in the elderly population, may be poorly assessed clinically^30^ and if severe, is a significant risk factor for post‐operative mortality,^31^ and therefore, the ability to identify haemodynamically significant valve lesions is an essential perioperative consideration.

A large proportion of patients had imaging of insufficient quality for assessment of valvular disease, and two cases of clinically important aortic stenosis were missed by the junior doctor performing the ultrasound. The correct diagnosis using ultrasound requires both image acquisition and image interpretation, both of which are operator dependent. Compared with an expert sonographer using a high‐end cart‐based ultrasound machine performing comprehensive ultrasound examination, it is expected that junior doctors using hand‐carried ultrasound devices will miss important pathology. This was demonstrated by Kobal *et al*.^32^ who investigated the accuracy of cardiovascular findings conducted by medical students with limited training and a hand‐carried ultrasound, vs. board‐certified cardiologists using clinical examination alone, with the gold standard of a sonographer with a high‐end cart‐based ultrasound machine. Compared with the sonographer, the medical students correctly identified pathology in 75%, whereas the cardiologists correctly identified pathology in only 49%. This illustrates that while pathology may be missed using point‐of‐care ultrasound, this is much less so compared with clinical examination alone.

For assessment of haemodynamic state, we demonstrated poor strength of agreement between the junior doctor and the expert's interpretation. The majority of poor agreement was due to misinterpretation of normal haemodynamic state as abnormal by the junior doctor. The reasons for this are unclear but might reveal a junior doctor's tendency to over‐diagnose when there is uncertainty, especially when faced with borderline cases or cases with inadequate imaging quality.

Our study identified a delay in surgery in seven patients following ultrasound examination. In all seven patients, this delay was the result of awaiting pre‐operative formal TTE or consultations by other inpatient units. However, ultrasound in this study uncovered a large proportion of patients with potentially serious cardiac pathology and the inconvenience or expense in generation of extra formal TTEs and surgical delay may be acceptable if it improves patient outcomes. While this was not investigated in our study, this likely warrants further research in the future. Furthermore, all ultrasound examinations in this study were performed by a single sonographer from the research team, and therefore, the timing of the ultrasound is limited by the sonographer's availability. However, if junior doctors were more widely trained in point‐of‐care ultrasound then it could be performed on admission by the admitting surgical team as part of routine assessment allowing for more time for definitive investigations and consultations with relevant inpatients units where necessary.

### Limitations

The current study is limited by its observational design, and its inability to assess whether changes to diagnosis and management improves patient outcomes. Nevertheless, the goal of this study was mainly to establish a proof of concept. Secondly, in this study we assessed the quality of junior doctor‐performed image interpretation based on the stored images and the large number of excluded studies due to poor image quality limits our ability to draw meaningful conclusions regarding accuracy. Therefore, it is important for a novice sonographer to recognise when image quality is too poor for diagnosis and we advise that a lead‐in phase of training and verification will be important for future trials investigating the utility of point‐of‐care ultrasound in clinical practice. Thirdly, larger‐scale studies are warranted to reproduce the findings reported in this study. Furthermore, our study is limited by the use of a single sonographer and a single expert reviewer and future studies could benefit from more than just one junior doctor and one expert to serve as comparators. Lastly, the primary method of communicating the ultrasound results to the treating team was *via* a written report, and while the sonographer was requested not to offer management advice, verbal discussion of the findings was not precluded.

## Conclusion

A focused cardiac and lung ultrasound examination performed by a junior doctor on patients presenting for emergency non‐cardiac surgery can change pre‐operative diagnosis and management. The predominant effects of detecting abnormalities on ultrasound were changes in fluid therapy, involvement of other inpatient units in the perioperative optimisation of patients and ordering of imaging to confirm ultrasound findings.

## Authorship statement

We acknowledge that the authorship listing conforms with the journal's authorship policy and that all authors are in agreement with the content of the submitted manuscript.

## Funding

No funding information is provided.

## Conflict of Interest

None to declare.

## Supporting information


**Appendix S1.** Diagnosis and management form.

**Appendix S2.** Image quality scoring system.

**Appendix S3.** Background paper on image quality scoring system.

**Table S1.** Ultrasound training of the junior doctor performing focused ultrasound.

**Table S2.** Preoperative clinical diagnoses, ultrasound findings and management changes in patients that had significant findings on point‐of‐care ultrasound.

**Figure S1.** Ultrasound report form.

**Figure S2.** Study process.
