# Supplementary material for: Impact of focused cardiac and lung ultrasound screening performed by a junior doctor during admission to the surgical ward on patients before emergency non‐cardiac surgery: A pilot prospective observational study
Source: Australas J Ultrasound Med. 2022 Oct 13;26(2):75–84. doi: 10.1002/ajum.12321 (PMC10225004; doi:10.1002/ajum.12321)
Supplement: Supplementary file 1 — Appendix S1. Diagnosis and management form. [file AJUM-26-75-s003.docx]

# Clinical Assessment

After conducting your clinical assessment and reviewing available pathology and imaging, please complete the following diagnosis and management plans

| Date of Assessment | DD/MM/YY |  |
| --- | --- | --- |
| Variable | Definition | Enter Value |
| Assessing Doctor Seniority | 1 = consultant, 2 = senior registrar/fellow, 3 = junior registrar |  |
| Cardiovascular Ventricle |  |  |
| Ventricular function (most severe diagnosis) | 1 = normal, 2 = hypovolemia (empty), 3 = LV systolic heart failure, 4 = LV diastolic heart failure, 5 = LV systolic and diastolic heart failure, 6 = RV heart failure, 7 = vasodilation, 8 = Heart failure – but unsure of what type of heart failure |  |
| Ventricular function (second – less severe diagnosis if present) | 2 = hypovolemia (empty), 3 = LV systolic heart failure, 4 = LV diastolic heart failure, 5 = LV systolic and diastolic heart failure, 6 = RV heart failure, 7 = vasodilation, 8 = Heart failure – but unsure of what type of heart failure |  |
| Valve lesions |  |  |
| Moderate or severe valve abnormality (most severe lesion) | 1 = nil, 2 = AS, 3 = AR, 4 = MR, 5 = MS, 6 = TR, 7 = TS, 8 = PR, 9 = PS |  |
| Moderate or severe valve abnormality (second most severe lesion if present) | 1 = nil, 2 = AS, 3 = AR, 4 = MR, 5 = MS, 6 = TR, 7 = TS, 8 = PR, 9 = PS |  |
| Moderate or severe valve abnormality (third most severe lesion if present) | 1 = nil, 2 = AS, 3 = AR, 4 = MR, 5 = MS, 6 = TR, 7 = TS, 8 = PR, 9 = PS |  |
| Other cardiovascular disorder |  |  |
| Pulmonary embolus | 1= nil, 2 = yes |  |
| Cardiac tamponade or hemodynamic significant pericardial effusion | 1= nil, 2 = yes |  |
| Arrhythmia contributing to hemodynamic instability | 1 = nil, 2 = rapid supraventricular (AF/SVT), 3 = rapid ventricular (VT), 4 = atrial bradycardia, 5 = complete heart block |  |
| Lung pathology | All investigation results may be used to assist in the following lung pathology diagnoses, e.g., chest X-ray, CT scan |  |
| Left side (most severe pathology) | 1 = nil, 2 = pleural effusion, 3 = pulmonary oedema, 4 = collapse (minor consolidation or atelectasis), 5 = major consolidation, 6 = pneumothorax |  |
| Left side (second pathology) | 2 = pleural effusion, 3 = pulmonary oedema, 4 = collapse (minor consolidation or atelectasis), 5 = major consolidation, 6 = pneumothorax |  |
| Left side (third pathology) | 2 = pleural effusion, 3 = pulmonary oedema, 4 = collapse (minor consolidation or atelectasis), 5 = major consolidation, 6 = pneumothorax |  |
| Right side (most severe pathology) | 1 = nil, 2 = pleural effusion, 3 = pulmonary oedema, 4 = collapse (minor consolidation or atelectasis), 5 = major consolidation, 6 = pneumothorax |  |
| Right side (second pathology) | 2 = pleural effusion, 3 = pulmonary oedema, 4 = collapse (minor consolidation or atelectasis), 5 = major consolidation, 6 = pneumothorax |  |
| Right side (third pathology) | 2 = pleural effusion, 3 = pulmonary oedema, 4 = collapse (minor consolidation or atelectasis), 5 = major consolidation, 6 = pneumothorax |  |
| Is there evidence of obstructive airways disease (such as wheezing) | 1 = nil, 2 = yes |  |

AF, atrial fibrillation; AR, aortic regurgitation; AS, aortic stenosis; CT, computer tomography; LV, left ventricle; MR, mitral regurgitation; MS, mitral stenosis; PR, pulmonary regurgitation; PS, pulmonary stenosis; RV, right ventricle; SVT, supraventricular tachycardia; TR, tricuspid regurgitation; TS, tricuspid stenosis; VT, ventricular tachycardia

# Management Plan – BEFORE ULTRASOUND

Please document your planned management below

| Date of management plan | DD/MM/YY |  |
| --- | --- | --- |
| Management | Definition | Enter value |
| Surgery planned | 1 = immediately, 2 = within 24 hours, 3 = next available (emergency list), 4 = as an outpatient, 5 = unsure – admit for tests |  |
| Investigations |  |  |
| Blood tests | 1 = no, 2 = yes |  |
| Imaging – heart | 1 = no, 2 = yes |  |
|  | If yes, please specify |  |
| Imaging – lungs | 1 = no, 2 = yes |  |
|  | If yes, please specify |  |
| Imaging – other | 1 = no, 2 = yes |  |
|  | If yes, please specify |  |
| Consultation – medical specialty | 1 = no, 2 = general medicine, 3 = cardiology, 4 = respiratory |  |
| Consultation – other | Please specify |  |
| Consultation – critical care | 1 = no, 2 = ICU, 3 = anesthesia |  |
| Treatment |  |  |
| Fluid replacement (bolus) | 1 = no, 2 = crystalloid, 3= colloid, 4 = blood |  |
| Fluid maintenance | 1 = no, 2 = crystalloid, 3 = colloid, 4 = blood |  |
| Fluid – fluid restriction (not fasting for surgery) | 1 = no, 2 = yes |  |
| Fluid - diuretics | 1 = no, 2 = yes |  |

# Management Plan – AFTER ULTRASOUND

| Date of management plan | DD/MM/YY |  |
| --- | --- | --- |
| Management | Definition | Enter value |
| Surgery planned | 1 = immediately, 2 = within 24 hours, 3 = next available (emergency list), 4 = as an outpatient, 5 = unsure – admit for tests |  |
| Investigations |  |  |
| Blood tests | 1 = no, 2 = yes |  |
| Imaging – heart | 1 = no, 2 = yes |  |
|  | If yes, please specify |  |
| Imaging – lungs | 1 = no, 2 = yes |  |
|  | If yes, please specify |  |
| Imaging – other | 1 = no, 2 = yes |  |
|  | If yes, please specify |  |
| Consultation – medical specialty | 1 = no, 2 = general medicine, 3 = cardiology, 4 = respiratory |  |
| Consultation – other | Please specify |  |
| Consultation – critical care | 1 = no, 2 = ICU, 3 = anesthesia |  |
| Treatment |  |  |
| Fluid replacement (bolus) | 1 = no, 2 = crystalloid, 3= colloid, 4 = blood |  |
| Fluid maintenance | 1 = no, 2 = crystalloid, 3 = colloid, 4 = blood |  |
| Fluid – fluid restriction (not fasting for surgery) | 1 = no, 2 = yes |  |
| Fluid - diuretics | 1 = no, 2 = yes |  |
