# Supplementary material for: Impact of focused cardiac and lung ultrasound screening performed by a junior doctor during admission to the surgical ward on patients before emergency non‐cardiac surgery: A pilot prospective observational study
Source: Australas J Ultrasound Med. 2022 Oct 13;26(2):75–84. doi: 10.1002/ajum.12321 (PMC10225004; doi:10.1002/ajum.12321)
Supplement: Supplementary file 2 — Appendix S2. Image quality scoring system. [file AJUM-26-75-s004.pdf]

Parasternal long-axis view (10 points)

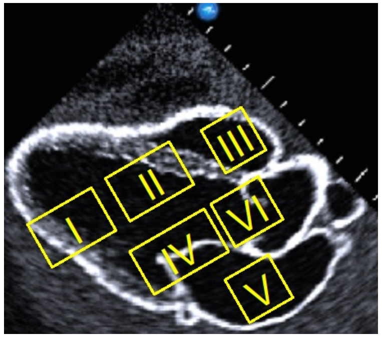

Structures
I – LV inferolateral border (at least 75% seen at end diastole)
II – LV anteroseptal border (at least 75% seen at end diastole)
III – Right ventricle (free wall should be seen)
IV – Mitral valve (both leaflet tips separating and coapting)
V – Left atrium (posterior wall should be seen)
VI – Aortic valve (both leaflet tips separating and coapting)

Other
VII – Depth setting adjusted so heart fills >3/4 of screen
VIII – LV not foreshortened (i.e. Apex should appear pointed, apex should contract towards centre of LV cavity and LV cavity should appear conical)
IX – Aorta fully open, including ascending aorta but the ascending aorta walls do not need to be seen clearly
X - Aortic and Mitral valves in the centre of image

Parasternal short-axis view, left ventricle (8 points)

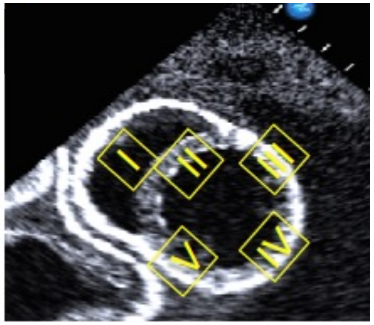

Structures
I – Right ventricle (at least 75% of endocardial border seen at end diastole)
II – Interventricular septum (at least 75% seen at end diastole)
III – LV anterior border (at least 75% seen at end diastole)
IV – LV lateral border (at least 75% seen at end diastole)
V – LV inferior border (at least 75% seen at end diastole)

Other
VI – LV cavity in centre of image (with part of RV visible)
VII – LV circular (vertical and horizontal dimensions within 20%) with no mitral structures visible
VIII – Papillary muscles fused to left ventricle during the entire cardiac cycle

Apical 4-chamber view (9 points)

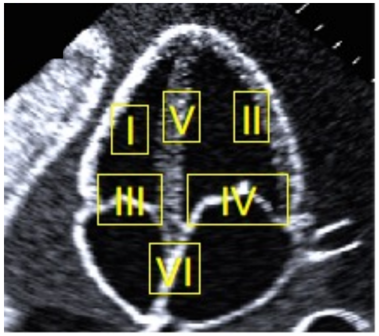

Structures
I – Right ventricle (at least 75% of endocardial border seen at end diastole)
II – LV lateral border (at least 75% seen at end diastole)
III – Tricuspid valve (both leaflet tips separating and coapting)
IV – Mitral valve (both leaflet tips separating and coapting)
V – Interventricular septum
VI – Interatrial septum (seen throughout systole and diastole)

Other
VII – Depth setting increased compared to parasternal views
VIII – Any part of the interventricular septum and interatrial septum in the centre of the screen
IX - No foreshortening of the left ventricle (i.e. Apex should appear pointed, apex should contract towards centre of LV cavity and LV cavity should appear conical)

Subcostal inferior vena cava view (4 points)

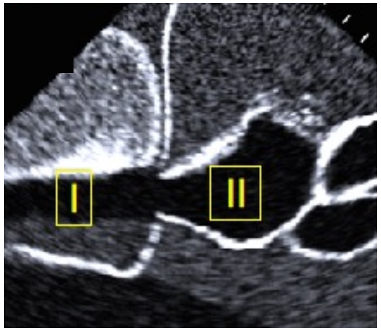

Structures
I – Inferior vena cava (walls parallel)
II – Right atrium

Other
III – Junction of inferior vena cava/right atrium in centre
IV – Inferior vena cava reaches edge of sector
