# Supplementary material for: Impact of focused cardiac and lung ultrasound screening performed by a junior doctor during admission to the surgical ward on patients before emergency non‐cardiac surgery: A pilot prospective observational study
Source: Australas J Ultrasound Med. 2022 Oct 13;26(2):75–84. doi: 10.1002/ajum.12321 (PMC10225004; doi:10.1002/ajum.12321)
Supplement: Supplementary file 3 — Appendix S3. Background paper on image quality scoring system. [file AJUM-26-75-s006.docx]

*Reprinted from Journal of Cardiovascular and Thoracic Anesthesia, Assessment of Image Quality of Repeated Limited Transthoracic Echocardiography After Cardiac Surgery, Vol 31, Canty DJ, Heiberg J, Tan JA, Yang Y, Royse AG, Royse CF, Mobeirek A, El Shaer F, Albacker T, Nazer R, Fouda M, Bakir B, Alsaddique A, Assessment of Image Quality of Repeated Limited Transthoracic Echocardiography After Cardiac Surgery, 965-972, Copyright 2016, with permission from Elsevier.*

**DETAILS, DEFINITIONS, AND EVALUATION OF THE IMAGE QUALITY SCORING SYSTEM**

The 68-point image quality scoring system assesses the two-dimensional appearance of standard cardiac structures from 10 standard views using the parasternal, apical, and subcostal windows. Each view comprises of between one and ten binary assessment questions, which assesses adequate visualization of cardiac structures, axis alignment e.g. absence of foreshortening of the LV or ascending aorta, positioning the region of interest in the center of the sector, and a suitable sector depth. The views included the parasternal long axis view (*10 points*), right ventricular inflow view (*4 points*), parasternal short axis view at the level of the aortic valve (*7 points*) and the mid LV (*8 points*), apical four-chamber view (*9 points*), apical five-chamber view (*1 point*), apical two-chamber view (*8 points*), apical long axis view (*9 points*), subcostal four-chamber view (*8 points*), and the subcostal inferior vena cava view (*4 points*). The total image quality score is the sum of points from each view expressed as a percentage of the maximum score (68 points). The score may also be expressed for each window and view, and may be applied to both the iHeartScan protocol^1^ as well as conventional comprehensive TTE protocols. A graphical illustration of the image quality scoring system is shown in Supplemental Material 4.

**Definitions**

For visualization of cardiac structures, the following definitions were used: *left ventricular borders,* *right ventricle and atriums*, at least 75% of endocardial border visible at end-diastole; *interatrial and interventricular septae*, visible throughout systole and diastole; *aortic, pulmonary, mitral, and tricuspid valves*, all leaflets separating and coapting.

*Foreshortening of the left ventricle* was defined as rounding of the apex, contraction of the apex towards the center of the left ventricular cavity, complete collapse of the left ventricular walls during systole, or missing cone-shape of the left ventricular cavity. *Circular left ventricle* in the parasternal short-axis view was defined as no more than 20% difference between any perpendicular diameters.

**Evaluation**

The image quality scoring system was evaluated prior to the study by performance of a standard TTE protocol by two expert echocardiographers on five normal adult volunteers. To simulate differing grades of image quality, each echocardiographer performed three TTE studies with differing image quality (best effort, good effort, and poor effort) on each of the five subjects. The images of the fifteen TTE studies were stored digitally in de-identified DICOM format. The order of the studies was randomized and assessed offline by two observers, who did not perform the TTE and who were blinded to both model and image quality effort.

Interobserver agreement was assessed by measuring the mean difference and 95% limits of agreement, and we considered the agreement between observers to be acceptable if the 95% limits of agreement were less than 30% of the mean value. The mean difference between observers was 6 ± 7% with limits of agreement that were 29% of the mean value, which was within the acceptable range. The ability of the image quality scoring system to discriminate between the differing grades of image quality was confirmed by separation of scores.

**REFERENCES**

**1.** Faris JG, Veltman MG, Royse CF: Limited transthoracic echocardiography assessment in anaesthesia and critical care. Best practice & research. Clinical anaesthesiology*.* 23:285-298, 2009.
