# Supplementary material for: Impact of focused cardiac and lung ultrasound screening performed by a junior doctor during admission to the surgical ward on patients before emergency non‐cardiac surgery: A pilot prospective observational study
Source: Australas J Ultrasound Med. 2022 Oct 13;26(2):75–84. doi: 10.1002/ajum.12321 (PMC10225004; doi:10.1002/ajum.12321)
Supplement: Supplementary file 4 — Table S1. Ultrasound training of the junior doctor performing focused ultrasound. [file AJUM-26-75-s002.docx]

**Table S1.** Ultrasound training of the junior doctor performing focused ultrasound

| **Coursework** |  |  |
| --- | --- | --- |
| *Course* | *Type* | *Details* |
| FCU - iHeartScan^TM^ Course | Supervised workshop | - Pre-reading including eLearning tutorials (25-30 hours) and 20 on-line interpretive e practice of pathology - 2-day supervised workshop including practice on a live human model and moderated discussion of pathologies - Post-workshop exam |
| FCU TTE Simulator Course | Semi-supervised Simulation^1^ | - Same pre-reading as above - 3-hour supervised workshop - 10 self-directed simulator cases of pathology - Post-workshop exam |
| FUSE Lung Ultrasound Simulator Course | Simulation^1^ | - Pre-reading including eLearning tutorials and 10 online case studies - 3-hour supervised workshop - 13 self-directed simulator cases - Post-workshop exam |
| **Non-coursework** |  |  |
| Supervised FCU and lung ultrasound examinations on hospital in-patients with pathology | Supervised learning | 30 FCU scans performed on patients with expert supervision and practice assessment of image quality and interpretation |

^1^The simulator used in the simulator courses was a Vimedix^TM^ simulator (CAE Healthcare, Montreal, Canada).
