# Supplementary material for: Impact of focused cardiac and lung ultrasound screening performed by a junior doctor during admission to the surgical ward on patients before emergency non‐cardiac surgery: A pilot prospective observational study
Source: Australas J Ultrasound Med. 2022 Oct 13;26(2):75–84. doi: 10.1002/ajum.12321 (PMC10225004; doi:10.1002/ajum.12321)
Supplement: Supplementary file 5 — Table S2. Preoperative clinical diagnoses, ultrasound findings and management changes in patients that had significant findings on point‐of‐care ultrasound. [file AJUM-26-75-s007.docx]

Table S2. Preoperative clinical diagnoses, ultrasound findings and management changes in patients that had significant findings on point-of-care ultrasound

| *Clinical Diagnosis* | *Ultrasound Findings* | *Management changes* |
| --- | --- | --- |
| No suspected cardiopulmonary diagnosis |  |  |
|  | Vasodilation | Fluids started |
|  | LV diastolic failure  RV failure | Formal outpatient TTE  Chest X-ray Cancelled |
|  | Aortic Stenosis | Formal outpatient TTE |
|  | Vasodilation | Fluids started |
|  | LV diastolic failure |  |
|  | LV systolic failure |  |
|  | LV systolic/diastolic failure  Mitral Stenosis  Aortic Stenosis | Formal outpatient TTE |
|  | LV systolic failure |  |
|  | LV diastolic failure |  |
|  | Tricuspid Regurgitation | Formal outpatient TTE |
|  | LV systolic/diastolic failure  Aortic Stenosis | General Medicine Consult  Anaesthetic Review  Surgery Cancelled |
|  | Aortic Stenosis | Formal outpatient TTE |
|  | LV diastolic failure  Tricuspid Regurgitation | Formal outpatient TTE |
|  | LV systolic/diastolic failure  Aortic Stenosis | Anaesthetic Review  Surgery Delayed |
|  | LV systolic/diastolic failure | Formal outpatient TTE  Chest X-ray Cancelled |
|  | LV diastolic failure  Tricuspid Regurgitation | Anaesthetic Review  Surgery Delayed |
|  | LV diastolic failure  Aortic Stenosis | Formal outpatient TTE  Anaesthetic Review  Fluids stopped |
|  | LV diastolic failure |  |
|  | LV diastolic failure  Mitral Stenosis  Aortic Stenosis  APO/interstitial syndrome | Formal inpatient TTE  Anaesthetic review  CXR Cancelled  Surgery delayed  Fluids stopped |
|  | LV diastolic failure | Formal outpatient TTE  Fluids stopped |
|  | LV systolic failure | Fluids stopped |
|  | LV systolic/diastolic failure  Mitral Regurgitation | Formal outpatient TTE  Anaesthetic Review  Fluids started |
|  | LV diastolic failure  Aortic Stenosis | Formal inpatient TTE  Anaesthetic review  Surgery delayed |
|  | Hypovolaemia | Fluids started |
|  | LV diastolic failure | Chest X-ray requested  Fluids started |
|  | LV diastolic failure | Formal outpatient TTE |
|  | LV systolic/diastolic failure | Formal inpatient TTE  Cardiology Consult  Chest X-ray requested  Surgery delayed  Fluids stopped |
|  | Aortic Stenosis | Formal inpatient TTE  Anaesthetic review  Surgery Delayed |
| Suspected Cardiopulmonary Diagnosis |  |  |
| Hypovolaemic/Vasodilation  Valvular Disease  Collapse/Atelectasis | LV systolic failure  Aortic Stenosis  APO/interstitial syndrome | Fluids stopped  Fluid restriction commenced  Diuretics commenced  Cardiology Consult |
| LV failure  Pulmonary oedema | LV systolic/diastolic failure  APO/interstitial syndrome  Bilateral Pleural Effusions | Diuretics commenced  Formal outpatient TTE  Cardiology Consult  Anaesthetic Review  Surgery Cancelled |
| RV failure  Valvular Disease | Aortic Stenosis | Fluids stopped  Anaesthetic Review |
| Valvular Disease | LV diastolic failure  Aortic Stenosis | Formal outpatient TTE  Anaesthetic review |
| Hypovolaemic/Vasodilated | APO/interstitial syndrome | Fluids stopped  Formal outpatient TTE |
| Valvular Disease | LV systolic/diastolic failure  Aortic Stenosis | Formal outpatient TTE  Anaesthetic Review |
| Hypovolaemic/Vasodilated | LV diastolic failure  RV failure | Formal inpatient TTE  Surgery delayed |
| LV failure | LV systolic/diastolic failure | Formal outpatient TTE |
| Hypovolaemic/Vasodilated | LV systolic/diastolic failure | Formal outpatient TTE |
| LV failure | LV systolic failure | Fluids stopped |
| LV failure  Pulmonary oedema | LV systolic/diastolic failure  Aortic Stenosis  Mitral Regurgitation  APO/interstitial syndrome | Fluids stopped  Fluid restriction commenced  Diuresis increased  Formal inpatient TTE  Cardiology Consult  Anaesthetic Review |
| LV failure | LV systolic failure  RV failure |  |
| Hypovolaemic/Vasodilated | LV systolic failure | Fluids stopped |

LV - Left ventricle, RV - Right ventricle, APO - Acute pulmonary oedema, TTE - Transthoracic Echocardiogram
