# Supplementary material for: Impact of focused cardiac and lung ultrasound screening performed by a junior doctor during admission to the surgical ward on patients before emergency non‐cardiac surgery: A pilot prospective observational study
Source: Australas J Ultrasound Med. 2022 Oct 13;26(2):75–84. doi: 10.1002/ajum.12321 (PMC10225004; doi:10.1002/ajum.12321)
Supplement: Supplementary file 6 — Figure S1. Ultrasound report form. [file AJUM-26-75-s001.pdf]

Patient Details

UR | **D.O.B.** dd/mm/yyyy | **Age** | Sex M

Surname | **First name**

Address

Suburb | State | Post Code

H: | M: | W:

Email:

Study Details

Exam ID | **Date** dd/mm/yyyy

Institution | **Operator**

☒ TTE ☐ TOE Quality ☐ Good ☐ Technically Difficult

Indication

Height | Weight | BSA | BMI

BP | HR | Rhythm

Ventricular Volume M-mode / 2D

☐ **Hypovolaemia** ☐ **Normal** ☐ **Dilated**

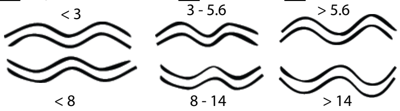

RV ☐ **Normal** ☐ **Increased**

Systolic Function

☐ **Increased** ☐ **Normal** ☐ **Decreased**

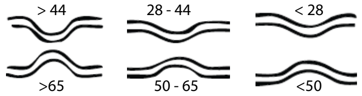

RV ☐ **Normal** ☐ **Decreased**

Ejection Fraction

LVEDD | LVEDA

LVESD | LVESA

FS | **EF/FAC**

Left Atrial Filling Pressure (Interatrial Septum Motion)

PSAX / A4Ch ☐ **Low LA Pressure** ☐ **Normal LA Pressure** ☐ **High LA Pressure**

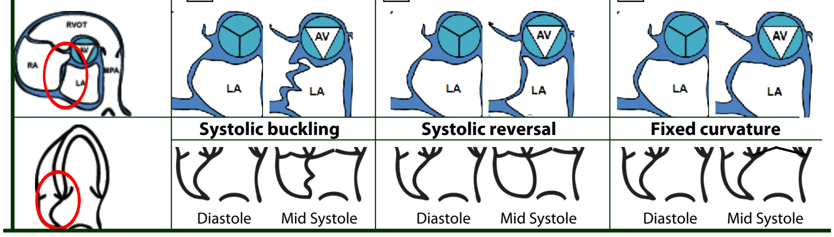

Valve Assessment

Examined	AV	MV	TV	PV
**Not Significant**	☐	☐	☐	☐
Haemodynamically Significant				
**Stenosis**	☐	☐	☐	☐
**Regurgitation**	☐	☐	☐	☐

☐ **Pericardial Effusion**

Haemodynamic State

	☐ Normal	☐ Empty	☐ Vaso dilated	☐ Primary Systolic Failure	☐ Primary Diastolic Failure	☐ Systolic & Diastolic Failure	☐ RV Failure
**Volume**	N	Decr	N	Incr	N / Decr	Incr	RV Incr
**Systolic Function**	N	N / Incr	Incr	Decr	N	Decr	RV Decr
**Filling Pressure**	N	Decr	N	N	Incr	Incr	Incr

iLungScan

RAnt	RPU	RPL		LAnt	LPU	LPL
☐	☐	☐	**Collapse**	☐	☐	☐
☐	☐	☐	**Consolidation**	☐	☐	☐
☐	☐	☐	**APO/Int. Syndr.**	☐	☐	☐
☐	☐	☐	**Pneumothorax**	☐	☐	☐
☐	☐	☐	**Effusion**	☐	☐	☐

cm | cm

mL | mL

Comments

Signature

iHeartScan™ iLungScan™

Haemodynamic **E**chocardiography **A**ssessment in **R**eal **T**ime

iHeartScan™ is a limited transthoracic echocardiography study that is qualitative and quantitative. It is intended to be completed in approximately 10 minutes. iLungScanTM is a focussed Lung Ultrasound

iHeartScan™ and iLungScan™ are registered trademarks. © Copyright 2013. All rights reserved. iHeartScan.com
