# Supplementary material for: Impact of focused cardiac and lung ultrasound screening performed by a junior doctor during admission to the surgical ward on patients before emergency non‐cardiac surgery: A pilot prospective observational study
Source: Australas J Ultrasound Med. 2022 Oct 13;26(2):75–84. doi: 10.1002/ajum.12321 (PMC10225004; doi:10.1002/ajum.12321)
Supplement: Supplementary file 7 — Figure S2. Study process. [file AJUM-26-75-s005.docx]

Figure S2. Study Process

Expert sonographer review of images for assessment of image acquisition and assessment

Updated management plan recorded by treating team

TTE – Transthoracic Echocardiogram, CT – Computer Tomography

Admitted to hospital, age ≥65 years and requiring emergency non-cardiac surgery

Focused Cardiac and Lung Ultrasound performed by junior doctor not involved in patient care

Changes to diagnosis recorded

Difference in management plan recorded as influence of ultrasound examination

Clinical diagnosis and management plan recorded by treating surgical team:

- Physical assessment

- Conventional Monitors

- Standard blood tests

- Chest X Ray

Informed patient (or carer) consent

Screened for exclusion criteria (No formal TTE, cardiac nuclear medicine scan or CT chest within 12 months)
